# Novel in vivo depiction of optic nerves hemorrhages in child abuse: a 3D-SWI pilot study

**DOI:** 10.1007/s00234-020-02622-6

**Published:** 2021-01-20

**Authors:** Giulio Zuccoli

**Affiliations:** Consultant for the Program for the Study of Neurodevelopment in Rare Disorders (NDRD), University of Pittsburgh, Children Hospital of Pittsburgh, 4401 Penn Ave, Pittsburgh, PA 15224 USA

**Keywords:** Optic nerves, Optic nerves hemorrhages, Abusive head trauma, Child abuse, Retinal hemorrhages, Forensic medicine, Infants, Brain MRI, Retinoschisis

## Abstract

**Purpose:**

Until now, the diagnosis of optic nerves hemorrhages in abusive head trauma (AHT) has been obtained only in the postmortem setting. The aim of the IRB-approved study was to assess the presence of optic nerves hemorrhages in AHT patients using 3D-SWI.

**Methods:**

Thirteen children with a final confirmed multidisciplinary diagnosis of AHT underwent coronal and axial 3D-SWI imaging of the orbits. The presence of optic nerve sheath (ONS) hemorrhages was defined by thickening and marked 3D-SWI hypointensity of the ONS, resulting in mass effect upon the CSF space. Optic nerve (ON) hemorrhages were defined by areas of susceptibility artifacts in the ON parenchyma. Superficial siderosis was defined by susceptibility artifact coating the ON. Furthermore, data about post-traumatic deformity of the ONS at the head of the optic nerve were collected.

**Results:**

The average age of the population was 7.9 ± 5.9 months old. The average GCS was 11.8 ± 4.5. The male to female ratio was 7:6. ONS hemorrhages were identified in 69.2% of cases. Superficial siderosis and ON hemorrhages were identified in 38.5 and 76.9% of cases, respectively. 3D-SWI also depicted traumatic deformity of the ONS at the level of the optic nerve head in 10 cases (76.9%). No statistical correlations were identified between RetCam findings and 3D-SWI findings or GCS and ON hemorrhages.

**Conclusion:**

This research shows that dedicated MRI with volumetric SWI of the orbits can depict hemorrhages in the ON, ONS, and ONS injury, in AHT victims.

## Introduction

The diagnosis of abusive head trauma is multidisciplinary [1]. AHT results from a combination of forces applied to the head of the child. These forces include shaking, blunt impact, throwing, or dropping the victim [2]. The forensic information provided by imaging and postmortem data to the diagnosis of AHT are substantial [1, 3–7]. However, gathering evidence, and overcoming alternative theories, especially in the court setting, remains challenging [8]. Thus, providing additional evidence of AHT, particularly when autopsy is not available, is critical. Until now, the diagnosis of optic nerve (ON) and optic nerve sheath (ONS) hemorrhages in AHT has been formulated only postmortem. This has been possible by using a technique which includes exenteration of the orbit soft tissues, optic nerves sectioning, gross pathological examination, and histological analysis. [9] The present study aims to identify hemorrhages within the ONS, and ON in AHT victims, by using 3D-SWI imaging of the orbits, with the same dedicated sequences of the orbits described elsewhere [10].

## Material and methods

This study was conducted in a single academic pediatric hospital. A group of 13 children with a final confirmed multidisciplinary diagnosis of AHT admitted at the Children Hospital of Pittsburgh between 2012 and 2015 were retrospectively analyzed as part of a larger IRB study regarding advanced imaging techniques, including 3D-SWI (IRB PRO10020141). All patients met the diagnostic criteria for AHT and underwent a standard brain MRI evaluation, with short and long TR imaging sequences. All patients underwent coronal and axial 3D-SWI imaging of the orbits, employing the 3D-SWI axial and coronal sequences used to identify retinal hemorrhages in prior studies, and the same MRI GE-based platforms [6, 10]. Lack of combined axial and coronal 3D-SWI imaging of the orbits represented exclusion criteria. Table 1 provides detailed information regarding the 3D-SWI sequences utilized in the present study. Sagittal and oblique reformats were obtained to confirm the presence of ON hemorrhages. Given the unavailability of neuroimaging-based literature regarding the diagnosis of ON hemorrhages in AHT, 3D-SWI were analyzed using as a reference of the neuropathological data from the postmortem literature [10–16]. Only one reader was chosen to analyze the images. One senior board-certified pediatric neuroradiologist, with more than 20 years of clinical experience in the field of pediatric neurotrauma, evaluated the images (GZ). The presence of ONS hemorrhages was defined by (a) ONS thickening and marked 3D-SWI hypointensity on both axial and coronal images confirmed by using the tool localizer available on the PACS workstation (iSite System, Philips), resulting in (b) mass effect upon the CSF space. ON hemorrhages were defined by the presence of any hemorrhage (very dark on 3D-SWI) involving the optic nerve parenchyma, on both axial and coronal images. Superficial siderosis was defined by scattered foci of susceptibility artifact (dark on SWI) along the surface of the optic nerve. Furthermore, data regarding post-traumatic deformity of the ONS, at the level of the optic nerve heads, as described by postmortem AHT studies were collected [9]. Deformities had to be confirmed on both axial and coronal 3D-SWI and were graded from 1 to 3, where grade 1 corresponds to mild deformity of the ONS without enlargement, grade 2 corresponds to deformity and mild enlargement of the ONS, and grade 3 corresponds to deformity with loss of the anatomical landmarks of the ONS with marked enlargement. Images were evaluated using a dynamic window setting tool, to better differentiate between fatty tissue and hemorrhage. The adapted Glasgow Coma Score and Glasgow Outcome Score indexes were collected for each patient, at discharge from the Hospital. RetCam reports were also collected. Normality of data was assessed by Kolmogorov-Smirnov test. Continuous numeric variables were expressed as mean ± SD or medians (IQR) and compared with a two-sample *t* test and one-way analysis of variance (for normally distributed data), or Mann-Whitney test and Kruskal-Wallis test (for non-normal distribution of data). Paired numeric variables were compared with the paired sample *t* test (for normally distributed data) or with the Wilcoxon signed-rank test (for non-normal distribution of data). Categorical variables were expressed as frequency number (%) and compared using *χ*2 test or Fisher’s exact test. All statistical analyses were performed using a SPSS version 20.0 (SPSS Inc., Chicago, IL), and a two-sided value of *P* < 0.05 was considered as statistically significant.

**Table 1:** 3D-SWI protocol

3D-SWI	TR/TE (ms)	Slice thick (mm)	FOV (mm)	Flip angle	Matrix	In-plane res. (mm)	*T*A (min)
AX 1.5 T	50.0/78.3	1	200	15	288 × 224	0.5	3.0
AX 3.0 T	46.6/26.0	1	200	15	320 × 256	0.6	3.3
COR1.5 T	53.1/26	1	180	15	320 × 256	0.6	6.0
COR 3.0 T	50/78.3	1	200	15	352 × 224	0.5	4.3

On Coronal SWI, the field of view included the orbits up to the coronal suture. This protocol was obtained in ~ 70% patients using a 1.5 T magnet and a 3.0 T magnet in the remainder 30%. *T*A acquisition time

## Results

Thirteen patients, 7 males (54%) and 6 females (46%), were identified (mean age, 7.9 months, min, 1.2 months; max, 20.8 months; Std. deviation, 7.9 months). The average GCS was 11.8 ± 4.5. The male to female ratio was 7:6. The outcome was good recovery, moderate disability, severe disability, and vegetative state in 38.5%, 30.8, 15.4, and 15.4%, respectively. Neuroimaging findings are reported in Table 2. ON hemorrhages were present in 76.9% cases; ONS were identified in 69.2% patients. Superficial siderosis and ONS deformity were noted in 38.5% and 76.9% patients, respectively.

**Table 2:** 3D-SWI results

	Yes (%)	No (%)
ONS hemorrhage	69.2	30.8
Superficial siderosis	38.5	61.5
Optic hemorrhages	76.9	23.1
ONS traumatic deformation*	76.9	23.1

Incidence of hemorrhagic lesions along the intra-orbital optic pathways. *ONS* optic nerve sheath; *at the level of the head of the optic nerve

**Fig. 1:**
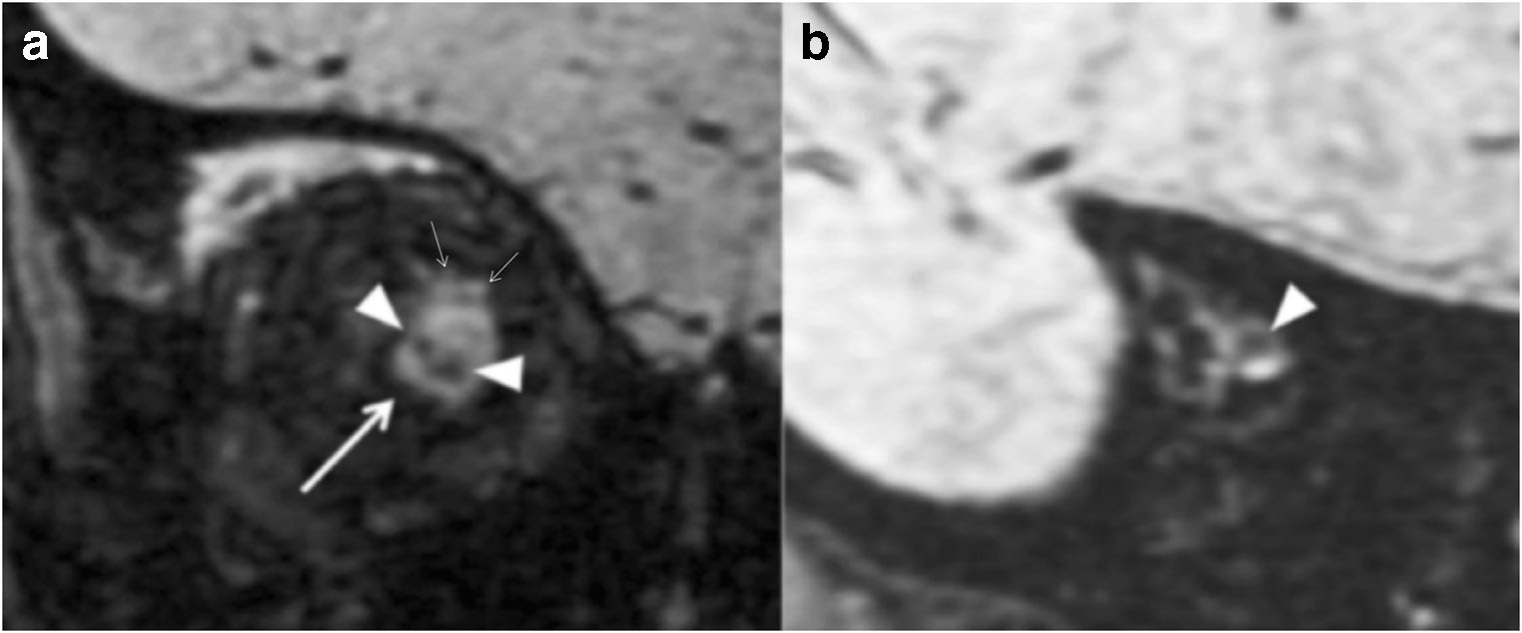
A 4.8-month-old patient. **a** Grade 3 deformity of the ONS at the optic nerve head level (left) with CSF leak (small arrows) and a subdural collection in the ONS (thicker arrow). A hemorrhagic lesion is identified within the inferior half of the right optic nerve (arrowheads). **b** ON hemorrhage at the optic fissure (arrowhead)

**Fig. 2:**
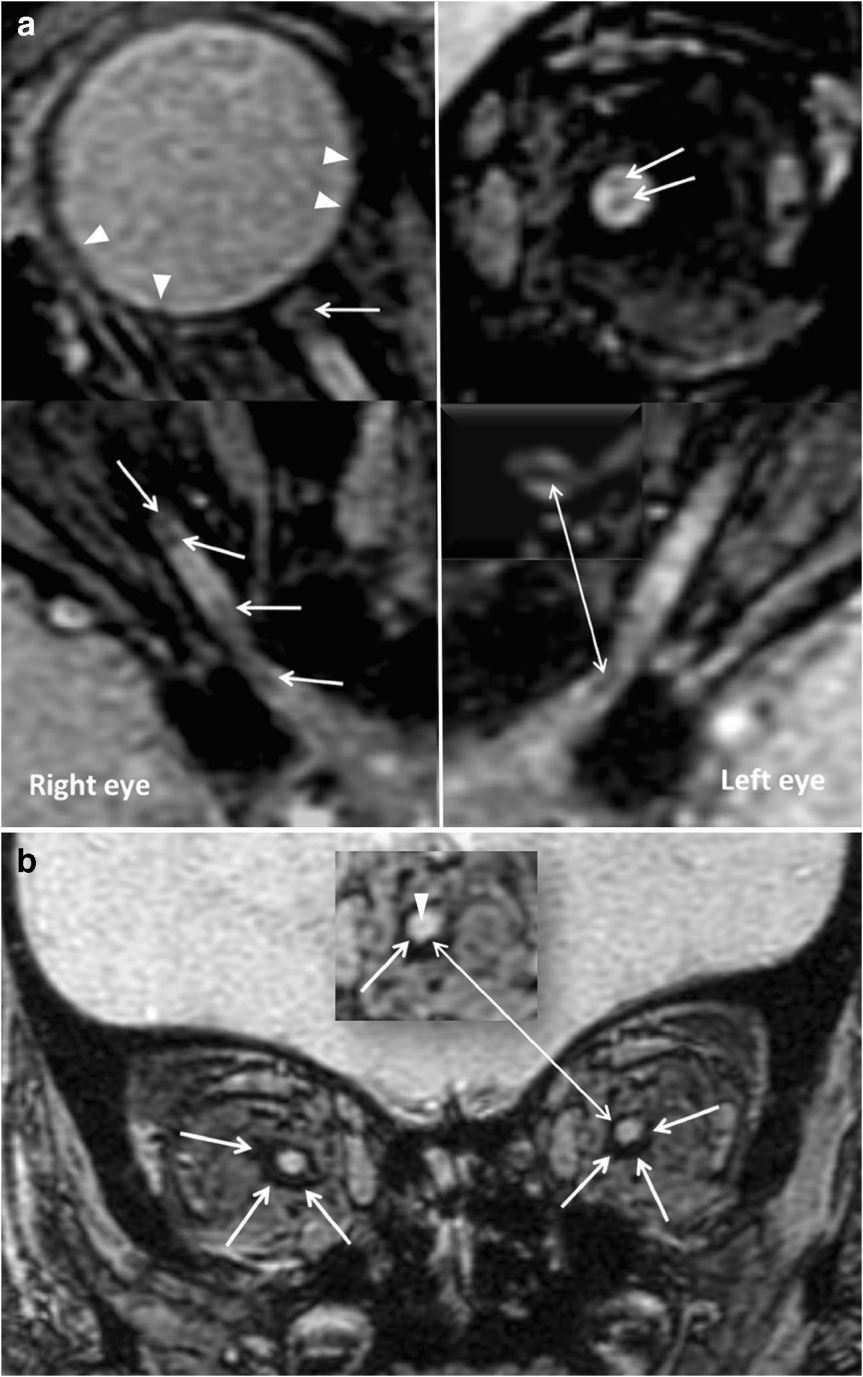
**a** A 11.7-month-old patient. A few ON hemorrhages are visible within the anterior (upper images) and posterior (bottom images) tracts of the ON (white arrows). The upper left image is a cropped image of the right globe, showing multiple retinal hemorrhages (arrowheads) and an ON hemorrhage near the right ON head (white arrow). The upper right image shows a coronal view of the anterior segment of the left ON, with two foci of ON hemorrhage (white arrows). **b** A coronal 3D-SWI from the same patient shows ONS hemorrhages (arrows) deforming the CSF space. Please note the presence of a fainted central hypointensity within the optic nerve corresponding to the central retinal vein (arrowhead). There is a perineural hemorrhagic infiltrate within the perineural fatty tissue, as demonstrated in the postmortem literature [9]

**Fig. 3:**
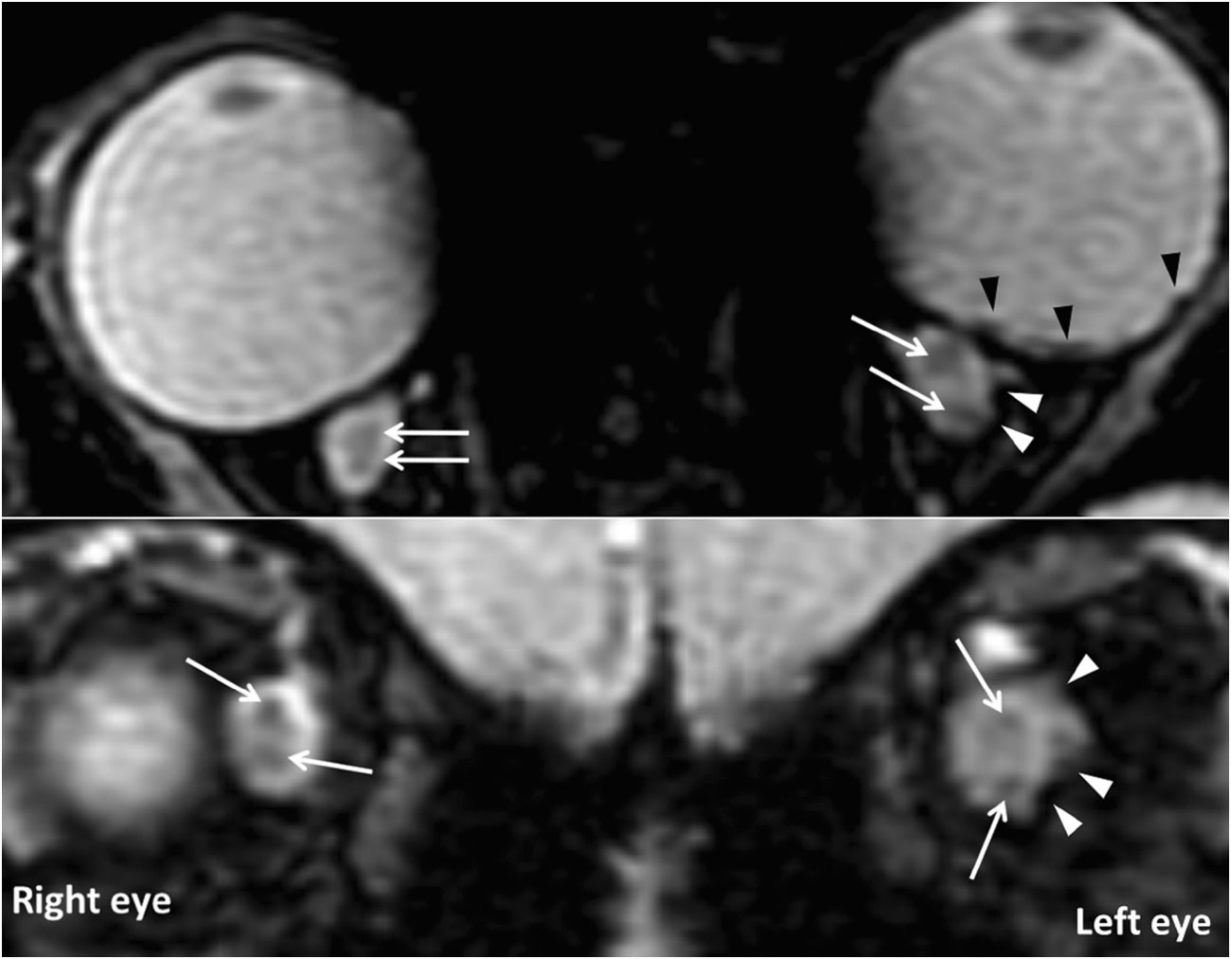
A 10.3-month-old patient with grade 3 deformity of the ONS showing ON hemorrhages, bilaterally (arrows), with hemorrhagic debris abutting the CSF space (white arrowheads) and retinal hemorrhages (dark arrowheads). Please note: the upper images are obtained in the axial plane, while the bottom images are obtained in the coronal plane right behind the globe

**Fig. 4:**
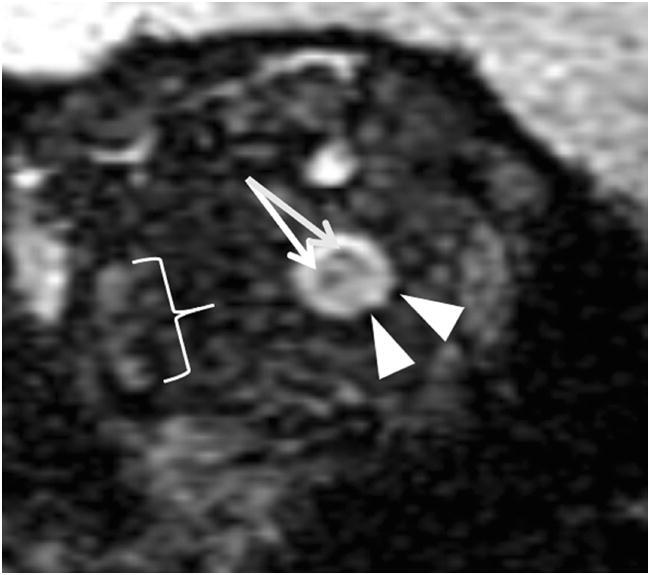
Right orbit of a 1.5-month-old patient with ON hemorrhages (arrows) and ONS hemorrhages (arrowheads). Additionally, there is hemorrhagic infiltration of the right lateral rectus muscle (bracket)

**Fig. 5:**
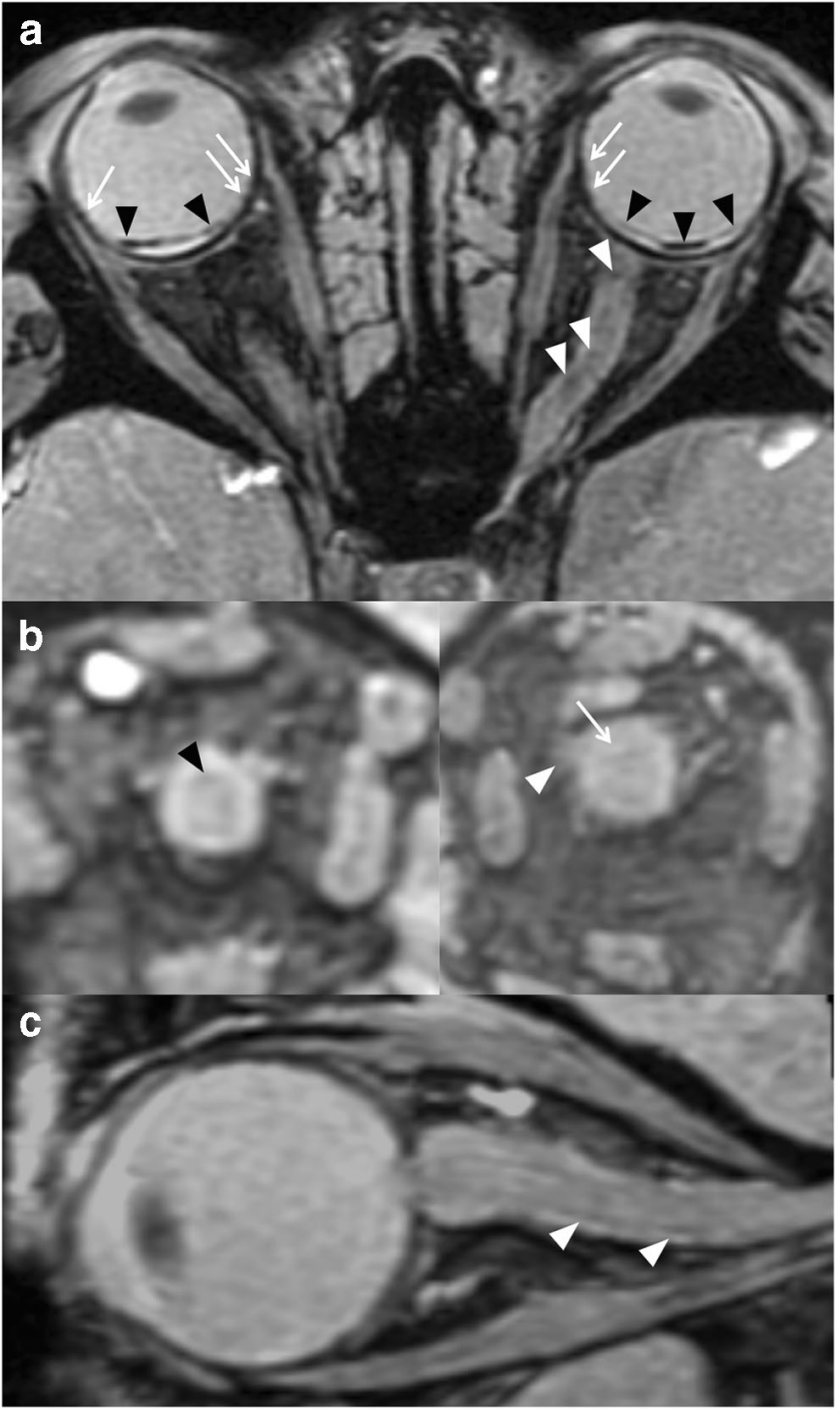
A 20.8-month-old patient. (**a**) Retinoschisis (black arrowheads) and retinal hemorrhages (white arrows) are seen. Scattered foci of superficial siderosis are seen along the left ON (white arrowheads). Coronal images (**b**) show mild superficial siderosis upon the right ON (black arrowhead) and a normal appearing left ON (arrow). Please note that the nerve is partially unmyelinated, as expected, showing a “light gray” signal. There is grade 2 deformity of the ONS on the left (white arrowheads). (**c**) Sagittal image of the right ON demonstrates small foci of superficial siderosis (white arrowheads). The nerve itself is otherwise normal

GCS and GOS were not statistically different between patients with and without ON/ONS hemorrhages, or traumatic deformity of the ONS. There was no statistical correlation between severity of deformity of the ONS and GCS, outcome, or ONS hemorrhages. Similarly, the presence of RetCam schisis was not significantly higher in patient with ON or ONS hemorrhages, and traumatic deformity of ONS. Figure 1, Fig. 2 a and b, Fig. 3, Fig. 4, and Fig. 5 reflect the spectrum of imaging findings encountered in the present MRI study.

## Discussion

ON hemorrhages in AHT were first described in a postmortem clinical report by Johnson and Coll. in 1986 [13]. On postmortem exam, the incidence of ON hemorrhages ranges from 65 to 100% [9, 14, 17]. The incidence of ONS hemorrhages is higher within the subdural space, followed in order of occurrence by the epidural, intradural, and subarachnoid space, respectively [9]. Similarly, in the present in vivo investigation, we observed a remarkably high incidence of ONS hemorrhages (69.2%) (Fig. 1, Fig. 2b), but still in the lower range, compared to the postmortem literature. This can be explained by the fact that none of the patients from this case series died from AHT. ON hemorrhages can be found within the different compartments of the intra-orbital optic pathways, including the subdural, epidural, subarachnoid, subpial, and intraoptic compartment [9, 12, 17, 18].

Postmortem, ON hemorrhages are better identifiable by using the microscope, on hematoxylin and eosin stain, although usually macroscopic examination is preferred [9, 12]. In this study, ON hemorrhages were identified in 76.9% of patients (Fig. 1, Fig. 2a, Fig. 3). ON hemorrhages did not correlate with GCS or GOS, which may be due to the small size of the population. The high incidence of ON hemorrhages observed in the present study may reflect the intrinsic fragility to trauma of the very tiny bridging veins abutting the central retinal vein. On the other hand, the visualization of ON hemorrhages is probably emphasized by the signal contrast between hemorrhage and unmyelinated ON on SWI [19]. Identifying the presence of ON hemorrhages and superficial siderosis can be clinically relevant from a prognostic point of view, especially in patients without cortical blindness, since sight impairment secondary to optic nerve degeneration is a known complication of AHT [20]. In this cohort, superficial siderosis was depicted in 38.5% of patients. In patients with superficial siderosis, iron and ferritin are responsible for triggering an immune response, resulting in neuroinflammation [21]. Therefore, the presence of superficial siderosis could lead to neurodegeneration and atrophy of the ON. Of note, superficial siderosis has been recently identified in the subarachnoid spaces of AHT victims [22, 23].

Furthermore, traumatic deformity of the ONS (Fig. 3) at the level of the coronal section passing through optic nerve head was clearly identified, a signature of AHT, described in the postmortem literature [9, 12, 18, 24]. This, when coupled with the presence of RH and ON hemorrhages, further supports the hypothesis that ON hemorrhages are traumatic in nature. From a mechanistic point of view, there could be a linear and/or angular acceleration threshold leading to traumatic rupture of the bridging veins abutting the central retinal vein, similarly to what has been postulated for the bridging veins of the brain by using a finite head model [25].

## Limitations

This is the first study of this nature; therefore, it needs to be validated by other groups. This study has no control patients; however, normal segments of the optic nerves of this AHT cohort were used as a reference for normal appearing nerve tissue (Fig. 5). Intracranial findings were not analyzed; however, the hypothesis that optic nerves bleeds are interconnected with intracranial hemorrhages has been dismissed by prior studies which have shown that there is no relationship between location of the intracranial hemorrhages and ON hemorrhages [9]. Furthermore, this would not explain the presence of ON hemorrhages as shown by hematoxylin eosin reports [12] and by the present study, since the ON compartment is not connected to the intracranial compartment, via the subarachnoid space [26]. No correlation was found between modified Glasgow Coma Score at discharge and ON hemorrhages, which makes unlikely a cause-effect between increased intracranial pressure and 3D-SWI findings [27]. This is also indirectly supported by the AHT literature in children with increased ICP, where no correlation is identified between increased ICP and retinal hemorrhages [28]. This study was not designed to identify the presence of fatty tissue or extraocular muscles hematomas, although hemorrhages of the perineural fatty tissue (Fig. 2b) and extraocular muscles (Fig. 4) were incidentally observed, which may open the door to future studies, using similar technical approaches. This is also related to the remarkable retrobulbar fatty signal variability on 3D-SWI.

## Conclusion

3D-SWI depicts ON and ONS hemorrhages in AHT, which has been, until now, exclusively a postmortem diagnosis. Furthermore, 3D-SWI depicts traumatic injury to the ONS. Despite the several limitations of this study, the in vivo identification of ON and ONS hemorrhages and ONS injury in AHT victims may help the multidisciplinary child protection team in reaching a more accurate diagnosis. Furthermore, 3D-SWI may represent a new tool for the prognosis of visual impairment in AHT victims [20]. Adding a new diagnostic technique able to depict previously unidentifiable imaging findings critical to the multidisciplinary diagnosis of AHT is very much needed. Several groups are developing MRI research protocols on the optic pathways in AHT, but most of these efforts occur disjointly. To be more effective, we must find ways to work as a team. A multinational collaborative study on this topic, collecting imaging and clinical data from AHT and accidental trauma pediatric patients, would be welcome.

## Abbreviations

AHT: Abusive head trauma
ON: Optic nerve
ONS: Optic nerve sheath

## References

[1] Choudhary AK, Servaes S, Slovis TL, Palusci VJ, Hedlund GL, Narang SK, Moreno JA, Dias MS, Christian CW, Nelson MD, Silvera VM, Palasis S, Raissaki M, Rossi A, Offiah AC (2018). Consensus statement on abusive head trauma in infants and young children. Pediatr Radiol.

[2] Joyce T, Huecker MR (2020) Pediatric Abusive Head Trauma. StatPearls Publishing [internet]. Treasure Island (FL). https://www.ncbi.nlm.nih.gov/books/NBK499836/29763011

[3] Choudhary AK, Dies Suarez P, Binenbaum G, Guandalini M, Cain T, Adamsbaum C, Panuel M (2019). Consensus statement on abusive head trauma: additional endorsements. Pediatr Radiol.

[4] Choudhary AK (2020). Understanding the importance of spinal injury in abusive head trauma (AHT). Pediatr Radiol.

[5] Teixeira SR, Gonçalves FG, Servin CA, Mankad K, Zuccoli G (2018). Ocular and intracranial MR imaging findings in abusive head trauma. Top Magn Reson Imaging.

[6] Zuccoli G, Panigrahy A, Haldipur A, Willaman D, Squires J, Wolford J, Sylvester C, Mitchell E, Lope LA, Nischal KK, Berger RP (2013). Susceptibility weighted imaging depicts retinal hemorrhages in abusive head trauma. Neuroradiology.

[7] Case ME (2008). Inflicted traumatic brain injury in infants and young children. Brain Pathol.

[8] Cowley LE, Maguire S, Farewell DM, Quinn-Scoggins HD, Flynn MO, Kemp AM (2018). Factors influencing child protection professionals’ decision-making and multidisciplinary collaboration in suspected abusive head trauma cases: a qualitative study. Child Abus Negl.

[9] Wygnanski-Jaffe T, Levin AV, Shafiq A, Smith C, Enzenauer RW, Elder JE, Morin JD, Stephens D, Atenafu E (2006). Postmortem orbital findings in shaken baby syndrome. Am J Ophthalmol.

[10] Zuccoli G, Khan AS, Panigrahy A, Tamber MS (2017). In vivo demonstration of traumatic rupture of the bridging veins in abusive head trauma. Pediatr Neurol.

[11] Wygnanski-Jaffe T, Morad Y, Levin AV (2009). Pathology of retinal hemorrhage in abusive head trauma. Forensic Sci Med Pathol.

[12] Elner SG, Elner VM, Arnall M, Albert DM (1990). Ocular and associated systemic findings in suspected child abuse: a necropsy study. Arch Ophthalmol.

[13] Johnson TE, Hoyt CS (1986). Optic nerve sheath and retinal hemorrhages associated with the shaken baby syndrome. Arch Ophthalmol.

[14] Rao N, Smith RE, Choi JH, Xiaohu X, Kornblum RN (1988). Autopsy findings in the eyes of fourteen fatally abused children. Forensic Sci Int.

[15] Puanglumyai S, Lekawanvijit S (2017). The importance of optic nerve sheath hemorrhage as a postmortem finding in cases of fatal abusive head trauma: a 13-year study in a tertiary hospital. Forensic Sci Int.

[16] Breazzano MP, Unkrich KH, Barker-Griffith AE (2014). Clinicopathological findings in abusive head trauma: analysis of 110 infant autopsy eyes. Am J Ophthalmol.

[17] Budenz DL, Farber MG, Mirchandani HG, Park H, Rorke LB (1994). Ocular and optic nerve hemorrhages in abused infants with intracranial injuries. Ophthalmology.

[18] Munger CE, Peiffer RL, Bouldin TW, Kylstra JA, Thompson RL (1993). Ocular and associated neuropathologic observations in suspected whiplash shaken infant syndrome: a retrospective study of 12 cases. Am J Forensic Med Pathol.

[19] Tong KA, Ashwal S, Obenaus A, Nickerson JP, Kido D, Haacke EM (2008). Susceptibility-weighted MR imaging: a review of clinical applications in children. Am J Neuroradiol.

[20] Jones R, Al-Hayouti H, Oladiwura D et al (2020) Optic atrophy in children: current causes and diagnostic approach. Eur J Ophthalmol:112067211989937. 10.1177/1120672119899378 10.1177/1120672119899378 31910664

[21] Koeppen AH, Michael SC, Li D, Chen Z, Cusack MJ, Gibson WM, Petrocine SV, Qian J (2008). The pathology of superficial siderosis of the central nervous system. Acta Neuropathol.

[22] Hahnemann ML, Kinner S, Schweiger B, Bajanowski T, Karger B, Pfeiffer H, Wittschieber D (2015). Imaging of bridging vein thrombosis in infants with abusive head trauma: the “tadpole sign”. Eur Radiol.

[23] Yilmaz U, Körner H, Meyer S, Reith W (2015). Multifocal signal loss at bridging veins on susceptibility-weighted imaging in abusive head trauma. Clin Neuroradiol.

[24] Muller PJ, Deck JHN (1974). Intraocular and optic nerve sheath hemorrhage in cases of sudden intracranial hypertension. J Neurosurg.

[25] Migueis GFJ, Fernandes FAO, Ptak M, Ratajczak M, Alves de Sousa RJ (2019). Detection of bridging veins rupture and subdural haematoma onset using a finite element head model. Clin Biomech.

[26] Selhorst JB, Chen Y (2009). The optic nerve. Semin Neurol.

[27] Murphy S, Thomas NJ, Gertz SJ, Beca J, Luther JF, Bell MJ, Wisniewski SR, Hartman AL, Tasker RC, Investigators of the Approa (2017). Tripartite stratification of the Glasgow Coma Scale in children with severe traumatic brain injury and mortality: an analysis from a multi-center comparative effectiveness study. J Neurotrauma.

[28] Morad Y, Kim YM, Armstrong DC, Huyer D, Mian M, Levin AV (2002). Correlation between retinal abnormalities and intracranial abnormalities in the shaken baby syndrome. Am J Ophthalmol.

